# Selection of reference genes for gene expression studies in human neutrophils by real-time PCR

**DOI:** 10.1186/1471-2199-6-4

**Published:** 2005-02-18

**Authors:** Xiaozhu Zhang, Lily Ding, Andrew J Sandford

**Affiliations:** 1 The James Hogg iCAPTURE Centre for Cardiovascular and Pulmonary Research, St. Paul's Hospital, University of British Columbia, Vancouver, Canada

## Abstract

**Background:**

Reference genes, which are often referred to housekeeping genes, are frequently used to normalize mRNA levels between different samples. However the expression level of these genes may vary among tissues or cells, and may change under certain circumstances. Thus the selection of reference gene(s) is critical for gene expression studies. For this purpose, 10 commonly used housekeeping genes were investigated in isolated human neutrophils.

**Results:**

Initial screening of the expression pattern demonstrated that 3 of the 10 genes were expressed at very low levels in neutrophils and were excluded from further analysis. The range of expression stability of the other 7 genes was (from most stable to least stable): GNB2L1 (Guanine nucleotide binding protein, beta polypeptide 2-like 1), HPRT1 (Hypoxanthine phosphoribosyl transferase 1), RPL32 (ribosomal protein L32), ACTB (beta-actin), B2M (beta-2-microglobulin), GAPD (glyceraldehyde-3-phosphate dehydrogenase) and TBP (TATA-binding protein). Relative expression levels of the genes (from high to low) were: B2M, ACTB, GAPD, RPL32, GNB2L1, TBP, and HPRT1.

**Conclusion:**

Our data suggest that GNB2L1, HPRT1, RPL32, ACTB, and B2M may be suitable reference genes in gene expression studies of neutrophils.

## Background

Neutrophils are the most numerous granulocytes in blood and are responsible for the first line of host defence. However, neutrophils have frequently been implicated in the pathogenesis of many diseases because they can produce various cytokines, chemokines and other proinflammatory mediators [1,2]. Numerous studies have been performed on the mechanisms that regulate the bioactivity of neutrophils. Understanding patterns of expressed genes may provide insight into complex regulatory networks and help to identify genes implicated in diseases. Quantitative real time PCR is one of the most powerful quantification methods for gene expression analysis. Similar to other methods used in expression studies, data from samples are usually required to be normalized against a set of data or references to correct for the difference in the amount of starting materials. The genes used as references are often referred to as housekeeping genes, assuming that those genes are constitutively expressed in certain tissues and under certain circumstances. However, the literature shows that the expression levels of the so called "housekeeping genes" may vary in different tissues, different cell types, and different disease stages [3-6]. Therefore, the selection of the reference genes is critical for the interpretation of the expression data.

In this study, we investigated 10 commonly used housekeeping genes (Table 1), and found 5 genes could be preferential reference genes for gene expression studies in human neutrophils.

## Results

### RNA quality and quantity

**Figure 1:**
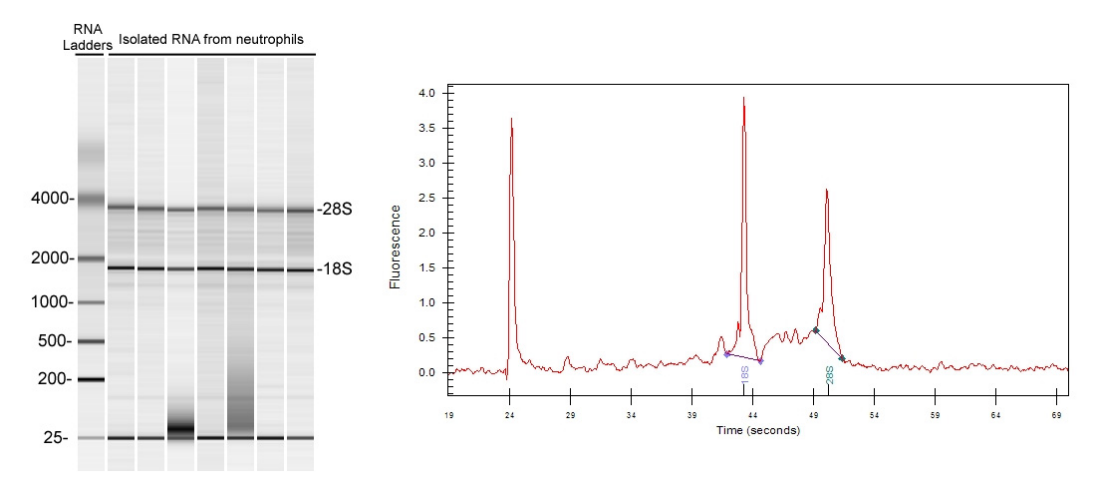
The results of RNA analysis by Agilent bioanalyzer. The first peak is a 20 bp molecular marker. The second and the third peaks are 18S and 28S rRNA.

RNA analysis by an Agilent 2100 Bioanalyzer provided the size profiles and the concentration of the samples. All the RNA samples used in this study were of good quality despite the long neutrophil isolation procedure. Intact rRNA subunits of 28S and 18S were observed on both the gel electrophoresis and electrophotogram, indicating that the degradation of the RNA was minimal (Figure 1).

### Expression patterns of the candidate genes in neutrophils

Initial screening for the gene expression pattern suggested that the 10 candidate housekeeping genes were differentially expressed in neutrophils (data not shown). Based on the band intensity of the PCR products, the two lowest expressed genes, two medium expressed genes and the three highest expressed genes were chosen for real-time PCR analysis. ABL1, PBGD and TUBB were excluded from further evaluation due to their extremely low expression level.

### Standard curve and real-time PCR

Standard curves were generated by using copy number vs. the threshold cycle (Ct). The linear correlation coefficient (R^2^) of all the seven genes ranged from 0.976 to 0.999. Based on the slopes of the standard curves, the amplification efficiencies of the standards were from 91%~100%, which were derived from the formula E = 10 ^1/-slope ^-1. The Ct values of all the 7 genes in all the unknown samples were within 15.9 to 33.5 cycles, covered by the range of the standard curves. Electrophoresis analysis of all the amplified products from real-time PCR showed a single band with the expected sizes, and no primer dimer was observed. The dissociation plots provided by the ABI Prism 7900HT also indicated a single peak in all the reactions.

### The stability and expression level of reference genes in the neutrophils

**Figure 2:**
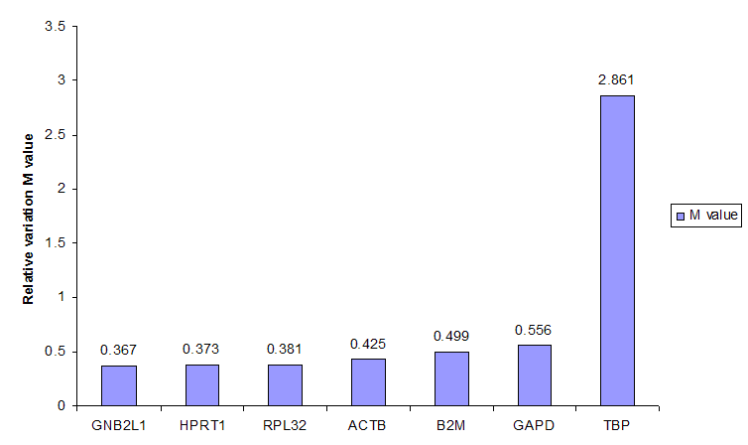
Gene expression stability of seven candidate reference genes in the neutrophil analyzed by the geNorm program. The threshold for eliminating a gene as unstable was *M *≥ 0.5.

The gene expression levels were measured by real-time PCR, and the expression stabilities were evaluated by the *M *value of GeNorm. The ranking of the expression stability in these genes was (from the most stable to the least stable): GNB2L1, HPRT1, RPL32, ACTB, B2M, GAPD and TBP (Figure 2). The M values of GNB2L1, HPRT1, RPL32, ACTB, and B2M were lower than 0.5, and therefore these genes were concluded to be stably expressed housekeeping genes in neutrophils.

**Figure 3:**
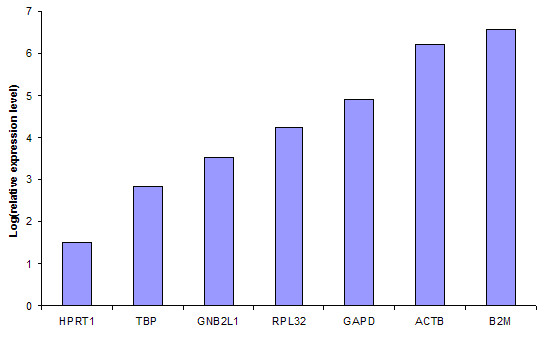
The relative expression level normalized against Normalization Factors from the 5 most stable genes (HPRT1, GNB2L1, RPL32, ACTB, and B2M) provided by geNorm. HPRT1 was the lowest expressed gene, and B2M was the highest among the candidate genes in neutrophils.

A normalization factor (NF) was calculated based on the geometric mean of the copy numbers of these 5 selected reference genes in each sample. After normalization against the NF, the ranking of the relative expression levels was (from high to low): B2M, ACTB, GAPD, RPL32, GNB2L1, TBP, and HPRT1 (Figure 3).

Based on both the expression stability and expression level, our data suggested that B2M and ACTB can be used as a reference gene for high abundance gene transcripts, RPL32 and GNB2L1 for medium abundance transcripts, and HPRT1 for low abundance transcripts in gene expression studies.

## Discussion

Real-time PCR is one of the most sensitive and flexible quantification methods for gene expression analysis. It provides simultaneous measurement of gene expression in many different samples for a number of genes. However, many factors in real-time PCR may affect the results, including the selection of the reference genes. An ideal reference gene should be expressed at a constant level among different tissues of an organism, at all stages of development, and should be unaffected by the experimental treatment. However, no one single gene is expressed at such a constant level in all these situations [4,7]. For example, ACTB, GAPD, 18S and 28S rRNA are the most commonly used reference genes, but a number of studies have provided solid evidence that their transcription levels vary significantly between different individuals, different cell types, different developmental stages, and different experimental conditions [3-6]. Therefore, thorough validation of candidate reference genes is critical for accurate analysis of gene expression.

It is also well known that RNA quality and quantity are critical for successful gene expression analysis. Degraded and inaccurately quantified RNA would give misleading results. In this study, the total RNA was extracted from isolated human neutrophils, and usually it takes 2–3 hours from drawing the blood to obtaining the pure neutrophils. RNA degradation is frequently observed. For this reason we performed careful RNA analysis by using an Agilent 2100 Bioanalyzer (Agilent Technologies) before the gene expression study. The results indicated our RNA samples were of good quality. Other quantification methods which need a microgram-level of RNA were not practical for our study because the amount of RNA extracted from the neutrophils from 10 ml blood was very limited (around 3–5 μg).

DNA contamination is another important factor that affects the accuracy of gene expression analysis. In this study, the following steps were taken to prevent and monitor DNA contamination: (1) RNase-free DNase I treatment on all the RNA samples; (2) The primers were designed to be able to distinguish the PCR product derived from mRNA or genomic DNA (Table 2); (3) Dissociation analysis by ABI Prism 7900HT; (4) Gel electrophoresis of all the amplified PCR products. With all these precautions in place we were confident that there was no detectable DNA contamination. The signal from SYBR I was specifically from the desired amplicons, not from artefacts (primer dimers or genomic DNA contamination).

For the reasons discussed above, we have confidence that our gene expression results were accurate and reliable, and we further analyzed the expression stability and expression level. The principle that the expression ratio of two ideal reference genes should be identical in all samples is well established. Based on this principle we found GNB2L1, HPRT1, RPL32, ACTB, and B2M were stably expressed in the neutrophils, and they were used for the calculation of a normalization factor (NF). After normalization we found B2M was the most highly expressed, followed by ACTB, RPL32, GNB2L1, and HPRT1 was the lowest expressed. As the expression level of the reference genes may be an additional factor for consideration in the process of reference gene selection, this ranking of the relative expression level of the candidate reference genes may be informative for future gene expression studies in neutrophils.

## Conclusion

To our knowledge, this is the first detailed study of the stability and level of reference gene expression in neutrophils. We found GNB2L1, HPRT1, RPL32, ACTB, and B2M are good choices for reference gene(s) selection. B2M and ACTB can be used for high-abundance mRNA, RPL32 and GNB2L1 for medium-abundance mRNA, and HPRT1 for low-abundance mRNA in expression studies of neutrophils. For more accurate normalization, as suggested by other authors [8], we recommend a combination of the stably expressed genes GNB2L1, HPRT1, RPL32, ACTB, and B2M as a panel of reference genes for the normalization.

## Methods

### Candidate genes for expression studies

Ten housekeeping genes were selected from commonly used reference genes (ABL1, ACTB, B2M, GAPD, GNB2L1, HRPT1, PBGD, RPL32, TBP, and TUBB). Gene symbols and their full names, gene accession numbers as well as functions are listed in Table 1. These genes were chosen because they have different functions in order to avoid genes belonging to the same biological pathways that may be co-regulated. In selecting the genes to be analyzed, preference was given to pseudogene-free genes in the NCBI linked database (Table 1). All the primers were designed by the software, Primer 3, . Hairpin structure and primer dimerization were analyzed by NetPrimer. Primers spanning at least one intron were chosen to minimize inaccuracies due to genomic DNA contamination. The length of the primers was from 18-mer to 22-mer, GC content was from 45% to 60%, and the expected PCR products range from 114 bp to 318 bp. If the genes have pseudogenes, primers were chosen according to the alignment results between the genes and the pseudogenes, so that the primers were unique to the genes and different from the pseudogenes (Table 2).

### Subjects and sample preparation

A total of 15 volunteers were recruited (Table 3). All participants signed an informed consent document. 20 ml of peripheral blood was taken into heparinized tubes. Neutrophil isolation was performed by a Dextran-Ficoll sedimentation and centrifugation method [9]. Briefly, 20 ml blood was mixed with 5% Dextran (100,000–20,000 k Da; Sigma) in RPMI (9:2). After 40 min sedimentation, the white blood cell rich plasma was transferred onto the top of 10 ml Ficoll (Pharmacia), and centrifuged at 2500 rpm for 15 min. The cell pellet contained the neutrophils. The contaminating erythrocytes were removed by hypotonic lysis. The isolated neutrophils were subject to Kimra staining and microscopic cell differential count. The purity of the neutrophils was calculated. Samples with more than 2% eosinophils were excluded from the study. Half of the isolated neutrophils were used for RNA isolation.

### RNA extraction and RT-PCR

Total RNA was isolated using RNeasy Mini Kit (Qiagen) as described by the manufacturer. Genomic DNA was eliminated by RNase-free DNase I digestion (Qiagen) during the isolation procedure. Isolated total RNA was analyzed on an Agilent 2100 Bioanalyzer using the RNA 6000 pico labchip Kit (Agilent Technologies). First strand cDNA synthesis was carried out with SuperScript RNase H^- ^Reverse Transcriptase (Invitrogen) and random primers (Invitrogen) in a total volume of 20 μl. Reverse transcription was performed at 37°C for 1 hour followed by 72°C for 15 min.

### Amplification of gene transcripts

To screen the basal expression patterns of the candidate genes in neutrophils, three randomly selected samples were tested by PCR with the ten primer pairs (Table 2).

The expression study was performed using a 384 well plate on an ABI Prism 7900HT Sequence Detection System (Applied Biosystems) with QuantiTect SYBR Green PCR Kit (Qiagen). The reactions were performed according to the manufacturer's instructions with minor modifications. The PCR program was initiated at 95°C for 10 min to activate *Taq *DNA polymerase, followed by 45 thermal cycles of 15 seconds at 94°C, 30 seconds at 58°C and 30 seconds at 72°C. Size analysis of the PCR products (dissociation analysis or meting curve analysis) was performed immediately after the real-time PCR. The temperature range used for the melting curve generation was from 60°C to 95°C. Each sample was analyzed in triplicate wells. In addition, all the reactions were further subject to electrophoresis on 2.5% agarose gels stained with ethidium bromide to confirm the expected PCR products.

### Standard curves

The amplified fragments from each primer pair were purified with QIAquick PCR purification Kit (Qiagen), and confirmed by DNA sequencing (University of British Columbia, NAPS Unit). The concentrations of the PCR products were quantified by a spectrophotometer (Perkin-Elmer Lambda 2 UV/VIS Spectrometer), which were further transformed to copy numbers based on the length and base composition of the PCR products. A ten-fold series dilution was made and 10 to 1,000,000 copies were used for generating standard curves in the real-time PCR, plotted as Ct values (cycle numbers of threshold or crossing points) versus logarithms of the given concentrations of the DNA templates.

### Determination of Gene stability and expression levels in human neutrophils

Gene stability was also evaluated using the geNorm software program [8]. Briefly, this approach relies on the principle that the expression ratio of two perfect reference genes would be identical in all samples in all experimental conditions or cell types. Variation in the expression ratios between different samples reflects the fact that one or both of the genes are not stably expressed. Therefore, increasing variation in this ratio corresponds to decreasing expression stability. The geNorm program can be used to calculate the gene expression stability measure (*M*), which is the mean pair-wise variation for a gene compared with all other tested control genes. Genes with higher *M *values have greater variation in expression. The stepwise exclusion of the gene with the highest *M *value allows the ranking of the tested genes according to their expression stability. The proposed threshold for eliminating a gene as unstable was *M *≥ 0.5. In the final analysis, genes with M value lower than 0.5 were considered as stably expressed genes, and were used for normalization factor (NF) calculation. Using the NF we calculated and ranked the expression level of all the seven genes in our samples.

## Abbreviations

ACTB, beta-actin; ALB1, Abelson murine leukemia viral oncogene homolog 1; B2M, beta-2-microglobulin; cDNA, complementary DNA; GAPDH, glyceraldehyde-3-phosphate dehydrogenase; GNB2L1 (Guanine nucleotide binding protein, beta polypeptide 2-like 1; HPRT1, Hypoxanthine phosphoribosyltransferase 1; NCBI, National Center for Biotechnology Information; PCR, polymerase chain reactions; PBGD, porphobilinogen deaminase; RPL32, ribosomal protein L32; RT-PCR, reverse transcription-polymerase chain reactions; TBP, TATA-binding protein; TUBB, beta-tubulin

## Authors' contributions

XZ performed all the experimental procedures and was the primary author of the manuscript. LD participated in the study design and data analysis. AS conceived of the study, participated in the study design and coordination. All authors read and approved the final manuscript.

## Figures and Tables

**Table 1:** 10 selected candidate housekeeping genes

Gene symbol	Gene Name	Accession Number		Function	Gene synonyms
				
		mRNA	genomic DNA		
ABL1	Abelson murine leukemia viral oncogene homolog	NM_007313	NT_035014	Cytoplasmic and nuclear protein tyrosine kinase	ABL, JTK7, p150, c-ABL, v-abl
ACTB	Beta-actin	NM_001101	NT_007819	Cytoskeletal structural protein	
B2M	Bata-2-microglobulin	NM_004048	NT_030828	Cytoskeletal protein involved in cell locomotion	
GAPD	Glyceraldehyde-3-phosphate dehydrogenase	NM_002046	NT_009759	Glycolytic enzyme	G3PD, GAPDH
GNB2L1	Guanine nucleotide binding protein, β-peptide 2-like 1	NM_006098	NT_077451	Involved in binding and anchorage of protein kinase C	H12.3, RACK1, Gnb2-rs1
HPRT1	Hypoxanthine phosphoribosyltransferase 1	NM_000194	NT_011786	Constitutively expressed at low levels, involved in the metabolic salvage of purines in mammals.	HPRT, HGPRT
PBGD	Porphobilinogen deaminase	NM_000190	NT_033899	Deficiency of porphobilinogen deaminase results in acute intermittent porphyria	HMBS, AIP, UPS
RPL32	Ribosomal protein L32	NM_000994	NT_005927	Member of the 80 different ribosome proteins	
TBP	TATA-binding protein	NM_003194	NT_007583	Involved in the activation of basal transcription from class II promoter	GTF2D, SCA17, TFIID, GTF2D1
TUBB	Beta-tubulin	NM_001069	NT_034880	Member of the tubulin family of structural proteins	

**Table 2:** Primers for Real-Time PCR

Gene symbol	Length	Position in cDNA		Sequence (5'-3')
ABL1	20	Exon 7	1217-1236	TGACAGGGGACACCTACACA
	20	Exon 9	1535-1516	TCAAAGGCTTGGTGGATTTC
ACTB	20	Exon 2	210-229	CATCGAGCACGGCATCGTCA
	21	Exon 3	420-400	TAGCACAGCCTGGATAGCAAC
B2M	19	Exon 2	268-287	ACTGAATTCACCCCCACTGA
	20	Exon 4	381-362	CCTCCATGATGCTGCTTACA
GAPD	20	Exon 7	728-747	TGGACCTGACCTGCCGTCTA
	22	Exon 8	970-948	CCCTGTTGCTGTAGCCAAATTC
GNB2L1	20	Exon 3	327-346	GAGTGTGGCCTTCTCCTCTG
	20	Exon 5	550-531	GCTTGCAGTTAGCCAGGTTC
HRPT1	20	Exon 4	322-341	GACCAGTCAACAGGGGACAT
	22	Exon 7	516-495	AACACTTCGTGGGGTCCTTTTC
PBGD	18	Exon 11-12	764-781	AGGATGGGCAACTGTACC
	20	Exon 13	995-976	GTTTTGGCTCCTTTGCTCAG
RPL32	19	Exon 1/2	33-51	CATCTCCTTCTCGGCATCA
	20	Exon 3	185-166	AACCCTGTTGTCAATGCCTC
TBP	20	Exon 4	623-642	GAACCACGGCACTGATTTTC
	20	Exon 5	780-761	CCCCACCATGTTCTGAATCT
TUBB	20	Exon 3	240-259	CTTCGGCCAGATCTTCAGAC
	20	Exon 4	416-397	AGAGAGTGGGTCAGCTGGAA

**Table 3:** Characteristics of the study subjects

	No.	Gender (F/M)	Age	Diagnosis		
				
				Allergy	Asthma	Healthy
Asian	6	4/2	33 ± 3	3	-	3
Caucasian	9	3/6	33 ± 9	3	2	4
